# Supplementary material for: Alternative and Complementary Therapies against Foodborne Salmonella Infections
Source: Antibiotics (Basel). 2021 Nov 26;10(12):1453. doi: 10.3390/antibiotics10121453 (PMC8698041; doi:10.3390/antibiotics10121453)
Supplement: Supplementary file 1 [file antibiotics-10-01453-s001.zip › antibiotics-1441128-supplementary.pdf]

Table S1. Antimicrobial activity of aqueous herbal plants against *Salmonella* sp.

Sample	Con.	*S. Typhimurium*			*S. Enteritidis*		
		min	Max	mean±SE	Min	Max	mean±SE
	50	38.0	45.0	41.67±2.03^{abc}	40.0	44.0	41.33±01.33^{ab}
	25	31.0	35.0	33.67±1.33^{defgh}	30.0	35.0	32.33±1.45^{defgh}
Thyme	12.5	27.0	30.0	28.33±0.88^{hijk}	25.0	30.0	28.33±1.66^{fghijk}
	50	34.0	40.0	37.0±1.73^{cdef}	38.0	40.0	39.0±0.58^{abc}
	25	28.0	35.0	31.0±2.08^{ghi}	25.0	38.0	33.3±4.17^{cdefg}
Rosemary	12.5	18.0	25.0	21.0±2.08^{m}	15.0	29.0	23.33±4.27^{jklm}
	50	38.0	40.0	39.33±0.67^{bcd}	29.0	35.0	31.33±1.86^{defghi}
	25	30.0	36.0	32.67±1.76^{efghi}	25.0	29.0	27.67±1.33^{fghijkl}
Genger	12.5	26.0	30.0	28.33±1.20^{hijk}	15.0	20.0	18.0±1.53^{mno}
	50	40.0	47.0	43.0±2.08^{ab}	40.0	48.0	44.0±2.31^{a}
Paprika	25	32.0	36.0	34.3±1.22^{defg}	35.0	37.0	36.0±0.58^{bcde}
	12.5	29.0	30.0	10.67±0.67^{ghij}	26.0	34.0	30.0±2.31^{efghij}
	50	32.0	37.0	15.67±1.45^{defg}	32.0	35.0	33.67±0.88^{cdef}
	25	18.0	25.0	21.0±2.08^{m}	25.0	27.0	26.0±0.58^{hijkl}
Black pepper	12.5	10.0	12.0	10.67±0.67^{nop}	20.0	24.0	21.3±1.33^{lmn}
	50	10.0	19.0	15.67±2.85^{n}	10.0	12.0	10.67±0.67^{pqr}
	25	9.0	15.0	12.0±1.73^{no}	8.0	9.0	8.67±0.33^{qr}
White pepper	12.5	5.0	7.0	5.67±0.67^{p}	5.0	5.0	5.0±5.55^{r}
	50	20.0	27.0	24.0±2.08^{klm}	25.0	30.0	28.0±1.53^{fghijkl}
	25	19.0	28.0	22.33±2.85^{lm}	20.0	25.0	21.67±1.6667^{klmn}
Latency	12.5	5.0	9.0	7.67±1.33^{op}	10.0	17.0	14.0±2.0817^{opq}
	50	40.0	45.0	42.33±1.45^{abc}	40.0	43.0	41.33±0.88^{ab}
	25	35.0	39.0	37.0±1.15^{cdef}	35.0	39.0	37.33±1.20^{bcd}
Bay leaf	12.5	19.0	25.0	21.33±0.19^{m}	20.0	29.0	24.07±2.60^{ijkl}
	50	10.0	19.0	14.67±26^{n}	10.0	20.0	15.0±.2.89^{opq}
	25	7.0	10.0	8.67±0.88^{op}	8.0	10.0	9.0±0.58^{qr}
Coriander	12.5	5.0	8.0	6.0±1.0^{p}	5.0	5.0	5.0±0.0^{r}
	50	45.0	47.0	46.3±0.67^{a}	40.0	46.0	43.67±1.56^{a}
	25	35.0	40.0	37.33±1.45^{cde}	30.0	40.0	36.33±3.18^{bcde}
Cinnamon	12.5	30.0	35.0	32.33±1.45^{efghi}	25.0	30.0	28.0±1.53^{fghijkl}
	50	22.0	30.0	27.33±2.67^{ijkl}	20.0	32.0	26.33±3.48^{hijkl}
	25	20.0	29.0	24.67±2.67^{jklm}	20.0	25.0	23.0±1.53^{klm}
Chili	12.5	5.0	10.0	8.0±1.53^{op}	10.0	12.0	10.67±0.67^{pqr}
	50	32.0	37.0	34.67±1.45^{defg}	20.0	32.0	27.33±3.72^{fghijkl}
	25	35.0	36.0	35.33±0.33^{defg}	20.0	26.0	23.67±1.86^{jklm}
Clove	12.5	20.0	29.0	24.67±2.60^{jklm}	5.0	15.0	10.0±2.89^{pqr}
	50	31.0	32.0	31.33±0.33^{fghi}	30.0	33.0	31.67±0.88^{defgh}
	25	22.0	25.0	23.67±0.88^{klm}	25.0	29.0	26.67±1.20^{ghijkl}
Turmeric	12.5	10.0	20.0	15.0±2.89^{n}	10.0	20.0	15.67±2.96^{nop}

Table S2. Antimicrobial activity of alcoholic herbal plants against salmonella.

Sample	*S. Typhimurium*				*S. Enteritidis*		
	Con.	Min	Max	Mean ±S.E.	Min	Max	Mean±S.E.
Black pepper	50	19	20	19.7 ± 0.33^{efg}	20	30	25.3 ± 2.9^{cd}
	25	15	18	$16.7\pm.88^{fgh}$	17	20	18 ± 1^{efg}
	12.5	5	8	7 ± 1^{lmn}	10	15	11.7 ± 1.7^{h}
White pepper	50	10	13	11.7 ± 0.88^{ijk}	17	20	$18.7\pm.9^{efg}$
	25	7	10	8.7 ± 0.88^{klmn}	5	6	5.3 ± 0.33^{j}
	12.5	5	5	$5\pm.0^{n}$	4	7	6.7 ± 0.9^{j}
Latency	50	20	25	22.3 ± 1.4^{de}	17	20	19 ± 1.0^{efg}
	25	16	19	$17.3\pm.88^{fgh}$	16	20	17.7 ± 1.2^{fg}
	12.5	6	15	11 ± 2.6^{jkl}	5	7	$6\pm.6^{j}$
Bay leaf	50	20	30	25 ± 2.9^{cd}	8	9	$8.7\pm.3^{hij}$
	25	15	19	16.7 ± 1.2^{fgh}	5	8	$6.3\pm.9^{j}$
	12.5	5	10	8 ± 1.5^{klmn}	5	5	$5\pm.00^{j}$
Coriander	50	15	20	17.33 ± 1.4^{fgh}	17	20	19 ± 1.0^{efg}
	25	15	16	$15.7\pm.3^{ghi}$	16	20	18.3 ± 1.2^{efg}
	12.5	7	8	$7.7\pm.33^{klmn}$	5	10	7.3 ± 1.4^{ij}
Cinnamon	50	36	40	37.7 ± 1.2^{a}	40	41	$40.33\pm.3^{a}$
	25	29	32	$30.7\pm.88^{b}$	31	37	33 ± 1.9^{b}
	12.5	21	30	27 ± 3.0^{bc}	18	20	$19\pm.67^{efg}$
Chili	50	19	22	$20.3\pm.88^{ef}$	20	24	22 ± 1.1^{de}
	25	12	19	15 ± 2.1^{hij}	15	18	$16.7\pm.9^{g}$
	12.5	10	10	$10\pm.0^{klm}$	15	18	16 ± 1.0^{g}
Clove	50	27	30	$28.7\pm.88^{bc}$	25	27	$26\pm.6^{c}$
	25	21	30	26 ± 2.6^{cd}	18	20	$19\pm.58^{efg}$
	12.5	9	10	$9.7\pm.33^{klm}$	8	16	11 ± 2.5^{hi}
Turmeric	50	10	10	$10\pm.0^{klm}$	20	24	21.3 ± 1.3^{ef}
	25	8	10	$9\pm.58^{klmn}$	15	18	$16.3\pm.9^{g}$
	12.5	5	7	$6\pm.58^{mn}$	5	10	7.3 ± 1.4^{ij}

Table S3. Antimicrobial activity of oil extracts against salmonella.

Sample	Con.	S. Typhimurium			S. Enteritidis		
		**Min**	**Max**	**Mean ± S E**	**Min**	**Max**	**Mean ± S E**
**Clove**	50	20.0	25.0	22.0 ± 1.53^{ijk}	20.0	25.0	22.67 ± 1.45^{efgh}
	25	17.0	20.0	18.33 ± 0.9^{klm}	20.0	23.0	$21.67 \pm .088^{fgh}$
	12.5	15.0	19.0	16.67 ± 1.2019^{lm}	19.0	20.0	$19.67 \pm .03^{fghi}$
**Garlic**	50	29.0	31.0	30.0 ± 0.58^{efg}	20.0	23.0	$21.67 \pm .09^{fgh}$
	25	23.0	30.0	26.67 ± 2.03^{ghi}	15.0	18.0	$16.67 \pm .09^{hi}$
	12.5	20.0	27.0	24.0 ± 2.08^{hij}	10.0	15.0	$12.33 \pm .14^{i}$
**Thyme**	50	31.0	35.0	32.67 ± 1.20^{ef}	20.0	26.0	$23.67 \pm .18^{efgh}$
	25	21.0	30.0	25.67 ± 2.60^{ghij}	20.0	27.0	$23.33 \pm .20^{efgh}$
	12.5	20.0	22.0	20.67 ± 0.67^{jkl}	20.0	24.0	$22.0 \pm .11^{fgh}$
**Rosemary**	50	28.0	31.0	29.67 ± 0.88^{efg}	21.0	23.0	$22.0 \pm .06^{fgh}$
	25	20.0	25.0	22.33 ± 1.45^{ijk}	15.0	20.0	$18.0 \pm .15^{ghi}$
	12.5	21.0	24.0	22.67 ± 0.88^{ijk}	10.0	12.0	$11.33 \pm .07^{i}$
**Mint**	50	45.0	46.0	45.67 ± 0.33^{b}	10.0	42.0	31.0 ± 1.05^{de}
	25	20.0	35.0	28.33 ± 4.41^{fgh}	25.0	30.0	$27.67 \pm .14^{ef}$
	12.5	11.0	20.0	15.33 ± 2.60^{m}	15.0	20.0	$17.33 \pm .14^{hi}$
**Cinnamon**	50	58.0	60.0	59.33 ± 0.67^{a}	54.0	60.0	$57.67 \pm .19^{a}$
	25	48.0	50.0	49.0 ± 0.5774^{b}	45.0	50.0	$46.67 \pm .17^{b}$
	12.5	35.0	40.0	38.0 ± 1.53^{cd}	38.0	40.0	$39. \pm .06^{c}$
**Nigella sativa**	50	50.0	52.0	50.67 ± 0.67^{b}	47.0	49.0	$48.0 \pm .06^{b}$
	25	39.0	42.0	40.33 ± 0.88^{c}	35.0	39.0	$36.67 \pm .12^{cd}$
	12.5	31.0	36.0	34.0 ± 1.53^{de}	25.0	28.0	$26.67 \pm .09^{efg}$

Table S4. Minimum inhibitory and minimum bactericidal concentrations (µg/ml) for drugs of choice against tested salmonella isolates.

Serotype	Isolate no.	Cefotaxime		Amoxicillin/ clavulanic acid		Ciprofloxacin	
		**MIC (µg/ml)**	**MBC (µg/ml)**	**MIC (µg/ml)**	**MBC (µg/ml)**	**MIC (µg/ml)**	**MBC (µg/ml)**
S. Typhimurium	1	0.25	0.5	16	32	8	16
	2	1	2	32	64	4	8
	3	4	8	32	64	64	64
S. Enteritidis	4	4	8	16	32	16	32
	5	0.5	0.5	16	32	32	64
	6	2	4	32	64	8	16

Table S5. TLC and GC-MS analyses of cinnamon oil extract.

Peak	RT	Name	Formula	Area	Area Sum %
1	17.739	Linalool	$C_{10}H_{18}O$	8904758.9	1.13
2	19.447	Camphor	$C_{10}H_{16}O$	37188281.92	4.73
3	22.105	(Z)-3-Phenylacrylaldehyde	C_9H_8O	13707295.51	1.74
4	24.733	2-Propenal, 3-phenyl-	C_9H_8O	726478635.2	92.39
